# Scaling law characteristics and spatiotemporal multicomponent analysis of syphilis from 2016 to 2022 in Zhejiang Province, China

**DOI:** 10.3389/fpubh.2023.1275551

**Published:** 2023-10-25

**Authors:** Haocheng Wu, Ming Xue, Chen Wu, Qinbao Lu, Zheyuan Ding, Xinyi Wang, Tianyin Fu, Ke Yang, Junfen Lin

**Affiliations:** ^1^Zhejiang Province Center for Disease Control and Prevention, Hangzhou, China; ^2^Key Laboratory for Vaccines and Prevention and Control of Infectious Disease of Zhejiang Province, Hangzhou, China; ^3^Hangzhou Centre for Disease Control and Prevention, Hangzhou, China

**Keywords:** syphilis, scaling law, multivariate time series model, joinpoint regression, epidemiology

## Abstract

**Background:**

Syphilis has caused epidemics for hundreds of years, and the global syphilis situation remains serious. The reported incidence rate of syphilis in Zhejiang Province has ranked first in the province in terms of notifiable infectious diseases for many years and is the highest in China. This study attempts to use the scaling law theory to study the relationship between population size and different types of syphilis epidemics, while also exploring the main driving factors affecting the incidence of syphilis in different regions.

**Methods:**

Data on syphilis cases and affected populations at the county level were obtained from the China Disease Control and Prevention Information System. The scaling relationship between different stages of syphilis and population size was explained by scaling law. The trend of the incidence from 2016 to 2022 was tested by the joinpoint regression. The index of distance between indices of simulation and observation (DISO) was applied to evaluate the overall performance of joinpoint regression model. Furthermore, a multivariate time series model was employed to identify the main driving components that affected the occurrence of syphilis at the county level. The *p* value less than 0.05 or confidence interval (CI) does not include 0 represented statistical significance for all the tests.

**Results:**

From 2016 to 2022, a total of 204,719 cases of syphilis were reported in Zhejiang Province, including 2 deaths, all of which were congenital syphilis. Latent syphilis accounted for 79.47% of total syphilis cases. The annual percent change (APCs) of all types of syphilis, including primary syphilis, secondary syphilis, tertiary syphilis, congenital syphilis and latent syphilis, were − 21.70% (*p* < 0.001, 95% CI: −26.70 to −16.30), −16.80% (*p* < 0.001, 95% CI: −20.30 to −13.30), −8.70% (*p* < 0.001, 95% CI: −11.30 to −6.00), −39.00% (*p* = 0.001, 95% CI: −49.30 to −26.60) and − 7.10% (*p* = 0.008, 95% CI: −11.20 to −2.80), respectively. The combined scaling exponents of primary syphilis, secondary syphilis, tertiary syphilis, congenital syphilis and latent syphilis based on the random effects model were 0.95 (95% CI: 0.88 to 1.01), 1.14 (95% CI: 1.12 to 1.16), 0.43 (95% CI: 0.37 to 0.49), 0.0264 (95% CI: −0.0047 to 0.0575) and 0.88 (95% CI: 0.82 to 0.93), respectively. The overall average effect values of the endemic component, spatiotemporal component and autoregressive component for all counties were 0.24, 0.035 and 0.72, respectively. The values of the autoregressive component for most counties were greater than 0.7. The endemic component of the top 10 counties with the highest values was greater than 0.34. Two counties with value of the spatiotemporal component higher than 0.1 were Xihu landscape county and Shengsi county. From 2016 to 2022, the endemic and autoregressive components of each county showed obvious seasonal changes.

**Conclusion:**

The scaling exponent had both temporal trend characteristics and significant heterogeneity in the association between each type of syphilis and population size. Primary syphilis and latent syphilis exhibited a linear pattern, secondary syphilis presented a superlinear pattern, and tertiary syphilis exhibited a sublinear pattern. This suggested that further prevention of infection and transmission among high-risk populations and improvement of diagnostic accuracy in underdeveloped areas is needed. The autoregressive components and the endemic components were the main driving factors that affected the occurrence of syphilis. Targeted prevention and control strategies must be developed based on the main driving modes of the epidemic in each county.

## Background

Syphilis is a chronic systemic infection caused by the subspecies of *Treponema pallidum* (*T. pallidum*) and is one of the classic sexually transmitted diseases. It can cause damage and pathological changes to almost all tissues and organs in the human body (1, 2). Syphilis is classified into primary syphilis, secondary syphilis, tertiary syphilis, latent syphilis, and congenital syphilis. In these classifications, primary, secondary, and tertiary syphilis are types of dominant syphilis, and latent syphilis has no clinical manifestations (3). On a global scale, syphilis has caused epidemics for hundreds of years. According to the WHO, approximately 7.1 million (2.4–11.5 million) people worldwide were newly infected with *T. pallidum* in 2020, and the global syphilis situation remains serious (4). Although early syphilis mainly causes damage to the skin and mucous membranes, late syphilis can cause damage to the nervous and cardiovascular systems, threaten life, and transmit syphilis to the foetus through the placenta, resulting in a series of adverse pregnancy outcomes, including spontaneous abortion, stillbirth, and congenital syphilis (5, 6). In addition, syphilis infection can promote the infection and transmission of HIV (7, 8). Since 2009, the reported incidence rate of syphilis in China has been the highest in terms of the incidence of Class B notifiable infectious diseases, making syphilis a major public health problem. The reported incidence rate of syphilis in Zhejiang Province has ranked first in the province in terms of notifiable infectious diseases for many years and is the highest in China. The rate before 2019 was higher than the national average, as well as that in surrounding areas such as Shandong, Jiangsu, Anhui, and Shanghai (9, 10). Syphilis remains a major public health concern for Zhejiang Province.

The occurrence of infectious diseases is influenced by many natural and social factors. Previous studies found that the occurrence of syphilis is related to various urban indicators, including *per capita* gross domestic product (GDP), population density, the number of health technicians, the proportion of people over 60 years of age, the proportion of males, and annual average temperature and precipitation (11, 12). However, the indicators included in different studies varied, and there were opposite effects on the same indicator, such as the number of medical institutions. The comprehensive impact of cities as a whole on the occurrence of syphilis remains unclear. The theory of complex urban systems provides a new perspective for understanding urban health (13, 14).

The city is a typical complex system with two meanings: firstly, the city itself is a complex system, and secondly, the urban system composed of multiple cities is also a complex system. A new research paradigm focuses on the common characteristics exhibited by cities under different histories, geographies, and cultures. The scaling law is one of the mechanisms under the theory of complex urban systems. The nonlinear relationship between urban indicators and population size is usually quantitatively characterized by scaling exponent (15–17). Many studies have reported a superlinear scaling relationship between the incidence of infectious diseases, including COVID-19, dengue fever, influenza, and acquired immunodeficiency syndrome (AIDS), and population size (18–21).

However, studies scaling relationship between syphilis incidence and population size and the scaling trend over time are scarcely. Therefore, this study employ the scaling law to explain the scaling relationship between syphilis incidence and population size in Zhejiang Province, China. Additionally, to identify the main driving components affecting the occurrence of syphilis in different regions of Zhejiang, a multivariate time series model was applied (22).

## Materials and methods

### Setting and area of study

This is an ecological study on syphilis of Zhejiang Province. Zhejiang Province is a provincial-level administrative region of the People’s Republic of China, with Hangzhou City as its capital. It is located on the southeast coast of China, spanning 27°02′ ~ 31°11′north latitude and 118°01′ ~ 123°10′east longitude. There are 11 cities in Zhejiang Province, and as of the end of 2022, the permanent population of Zhejiang Province is approximately 65 million people.

### Data collection

Data on syphilis diagnosed at medical institutions were collected between January 2016 and December 2022 from the China Disease Control and Prevention Information System. The population data at the county level (90 counties in total) were updated by the company responsible for system operation and maintenance, and the new population data were imported into the system every December. The incidence rate of syphilis was computed by the system and exported. The diagnosis of syphilis was based on the “Diagnostic criteria for syphilis” (Version 2007) and “Diagnostic criteria for syphilis” (Version 2018) (23, 24). The formula for calculating the incidence of acquired syphilis in Zhejiang Province among 2016–2022 is the number of reported cases in the period divided by the total average population in the year. The incidence of congenital syphilis in Zhejiang Province among 2016–2022 is the proportion of reported cases of congenital syphilis to the total number of live births every year.

### Scaling law and meta-analysis

The scaling law of an urban system reveals the scaling relationship between urban indicators and population size at the same time point, and its function form is a power function (13–16):

(1) $$Y=Y_{0}N^{\beta }$$

In Eq. 1, *Y* represents the urban indicator (such as the cumulative number of confirmed cases of syphilis in county-level units as of a certain year). *N* is the population size; *Y_0_* and *β* are parameters, where *β* is the scaling exponent. By taking both sides of Eq. 1 with a base logarithm of 10, we can obtain Eq. 2:

(2) $$\mathrm{lg}Y=\beta \times \mathrm{lg}N+\mathrm{lg}Y_{0}$$

Equation 2 is a linear function. After taking the logarithm of confirmed cases and population size, a linear function is used for fitting, and the slope *β* of the fitted line is the scaling exponent. Although there are other nonlinear fitting methods, linear fitting is widely used in scaling law fitting due to its simplicity and ease of operation (25, 26). According to the relationship between *β* and 1, the urban indicators can be divided into three types: ①The indicator exhibits a superlinear scale relationship with population size (*β* > 1), and the scaling exponent of this type is approximately 1.15. ②The indicator exhibits a sublinear scale relationship with population size (*β* < 1), and the scaling exponent is approximately 0.85. ③The scale is linearly proportional to the population size, and the scaling exponent “*β*” is usually approximately equal to 1 (17).

Fixed-effects and random-effects meta-analyses were used to calculate a combined index of *β* based on the single-scale index from 2016 to 2022. Inverse variance weighting was used for pooling. The heterogeneity of the scaling exponent in different years was tested to select the fixed effects model or random effects model (27). The scaling law analysis and meta-analysis of scale index was run by R Studio (version 1.2.5001). The *I^2^*-statistic is an indicator of heterogeneity, the larger the value, the stronger the heterogeneity, while the value more than 75% means high heterogeneity. The *p* value less than 0.05 represented statistical significance for heterogeneity of scale index in different years and the random effect result should be applied.

### Joinpoint regression

Joinpoint regression is also known as segmented regression. This model does not require the data sequence itself to show obvious trends. This method is increasingly being used to determine the degree of change in time series data. The objective indicator was APCs of each period segment, estimated according to the following formula:

(3) $$APCi=\left[\exp\left(\beta_{i}\right)-1\right]\times 100$$

where $\beta_{i}$ represents the slope of the period segment (28, 29).

The joinpoint regression model was used to examine the trend of the incidence of syphilis from 2016 to 2022 by Joinpoint software (version 4.5.0.1). The number of segmentation points is subjected to hypothesis testing by this software. The first step assumes that there is no segmentation point, which is *H_0_*. If *H_0_* is rejected, this analysis is used to test for statistical significance between 1 segmentation point and 2 segmentation points. The *p* value less than 0.05 represented statistical significance for the model selection. The *p* value less than 0.05 or confidence interval (CI) does not include 0 represented statistical significance for the trend test.

This new index DISO is a merge of different statistical metrics including correlation coefficient (CC), absolute error (AE), and root mean square error (RMSE) according to the distance between the simulated model and observed field in a three-dimension space coordinate system. This method is used to quantitatively evaluate the comprehensive accuracy of joinpoint regression model, which is based on the Euclidean distance and flexible determination of statistical metrics and their numbers from the Da Dao Zhi Jian concept (30–33). The formula is as follows:

(4) $$\mathrm{DISO}=\sqrt{{\left(\mathrm{CC}\_1\right)}^{2}+{\mathrm{NAE}}^{2}+{\mathrm{NRMSE}}^{2}}$$

where NAE and NRMSE are normalized by the averaged values of the observed time series (33). In the model, a smaller disco value indicates better overall performance, and vice versa.

### Multivariate time series model

The spatiotemporal multicomponent model is based on the measuring of the Poisson branching process of population migration, while incorporating seasonal effects, long-term trends and over discretization characteristics. This model is widely used in multi regional time series data analysis. Assuming the research area is divided into *I* blocks, $Y_{i,t}$ is the case count in region i=1,…,I at time t=1,…,T . The count $Y_{i,t}$ is formally assumed to follow a negative binomial distribution $Y_{it}/Y_{i,t-1}\sim NegBin\left(\mu_{it},\psi \right)i=1,\ldots ,I,t=1,\ldots ,T$ , with an additively decomposed mean.

(5) $$\mu_{it}=\nu_{it}e_{it}+\lambda_{it}Y_{i,t-1}+\phi_{it}\underset{j\neq i}{\sum }\omega_{ji}Y_{j,t-1},$$

where ψ is an over dispersion parameter such that the conditional variance of $Y_{it}$ is $\mu_{it}\left(1+\Psi \mu_{it}\right)$ . The Poisson distribution results in a special case if ψ =0. The first component $\nu_{it}e_{it}$ represents the endemic risk, which captures factors such as population, sociodemographic variables, long-term trends, seasonality, and the climate within the local area y. The endemic mean is proportional to an offset of known expected counts $e_{it}$ , typically reflecting the population at risk. As a district-specific measure of disease incidence, the population fraction $e_{it}$ is included as a multiplicative offset. Here, the population at the county level as a multiplicative offset was incorporated into the endemic component. The next two components were observation-driven epidemic components. The second component $\lambda_{it}Y_{i,t-1}$ is the time autocorrelation risk, which measures the impact of the past epidemic on the current incidence of infectious diseases. The third component $\phi_{it}\underset{j\neq i}{\sum }\omega_{ji}Y_{j,t-1}$ denotes the spatiotemporal characteristics capturing the transmission from other counties. Each parameter $\nu_{it}$ , $\lambda_{it}$ , and $\phi_{it}$ is a linear predictor of the form.

(6) $$\log\left(\cdot it\right)=\alpha^{\left(\cdot \right)}+b_{i}^{\left(\cdot \right)}+\beta^{{\left(\cdot \right)}^{T}}z_{it}^{\left(\cdot \right)},$$

where “.” is one of ν,λ,ϕ ; $\alpha^{\left(.\right)}$ are intercepts; and $b_{i}^{\left(\cdot \right)}$ denotes the random effects, which account for heterogeneity between districts. $z_{it}^{\left(\cdot \right)}$ are exogenous covariates, including time effects, and $\beta^{{\left(.\right)}^{T}}$ denotes the coefficient of $z_{it}^{\left(\cdot \right)}$ .

$\omega_{ji}$ is the spatial contiguity weights matrix, which describes the strength of transmission from region j to region i . There are usually three models of neighbourhood weights, including the first-order neighbourhood model, the power law model and the second-order neighbourhood model. The variance components are estimated by maximizing the approximated marginal likelihood obtained *via* Laplace’s approximation. The optimal model is selected through the Akaike information criterion (AIC), and the smaller the value is, the better the fitting effect of the model (22, 34, 35). The multivariate time series model was run by R Studio (version 1.2.5001).

## Results

### The prevalence of syphilis

From 2016 to 2022, a total of 204,719 cases of syphilis were reported in Zhejiang Province, including 2 deaths, all of which were cases of congenital syphilis. Among all reported types of syphilis, latent syphilis accounted for the majority, with a total of 162,680 reported cases, accounting for 79.47% of total syphilis cases. In general, the reported incidence rate of syphilis from 2016 to 2022 showed a downward trend, with annual reported incidence rates of 62.16/100,000, 64.07/100,000, 54.89/100,000, 53.53/100,000, 41.37/100,000, 39.45/100,000 and 35.23/100,000 people, respectively. In joinpoint regression analysis, all types of syphilis do not reject the null hypothesis that there is no breakpoint. The APC of the incidence rate of total syphilis cases was −9.60% (95% CI: −12.90 to −6.10). Consistent with the general trend of syphilis, the reported incidence rate of all types of syphilis showed a downward trend from 2016 to 2022. The APCs of all types of syphilis, including primary syphilis, secondary syphilis, tertiary syphilis, congenital syphilis and latent syphilis, were − 21.70% (95% CI: −26.70 to −16.30), −16.80% (95% CI: −20.30 to −13.30), −8.70% (95% CI: −11.30 to −6.00), −39.00% (95% CI: −49.30 to −26.60) and − 7.10% (95% CI: −11.20 to −2.80), respectively. The reported incidence rate of primary syphilis declined from the highest rate of 7.78/100,000 people in 2016 to 1.98/100,000 in 2022. The reported incidence rate of secondary syphilis declined from the highest rate of 8.83/100,000 people in 2016 to 3.21/100,000 in 2022. The reported incidence rate of tertiary syphilis declined from the highest rate of 0.49/100,000 people in 2016 to 0.28/100000 in 2022. The reported incidence rate of latent syphilis fluctuated, and the incidence rates from 2016 to 2022 were 44.97/100,000, 48.27/100,000, 42.84/100,000, 44.52/100,000, 34.88/100,000, 32.75/100,000, and 29.75/100,000 people, respectively. From 2016 to 2022, the number of reported cases of congenital syphilis in Zhejiang Province remained low, and 48 cases and 28 cases were reported in 2016 and 2017, respectively. The annual average number of cases of congenital syphilis in the following 5 years was less than 10, and the incidence rate fluctuated at 1.3 per 100,000 live births during these 5 years (Supplementary Box S1).

The CC values were larger than 0.90 for all types of syphilis. Most NAE values were smaller than 0.1, but the value of total syphilis and latent syphilis were close to 1. All of NRMSE values were smaller than 2. The DISO values of primary syphilis, secondary syphilis, tertiary syphilis and congenital syphilis were smaller than 0.15, however, the values of total syphilis and latent syphilis were close to 1.4 (Supplementary Box S2).

### Scaling law of syphilis

**Figure 1:**
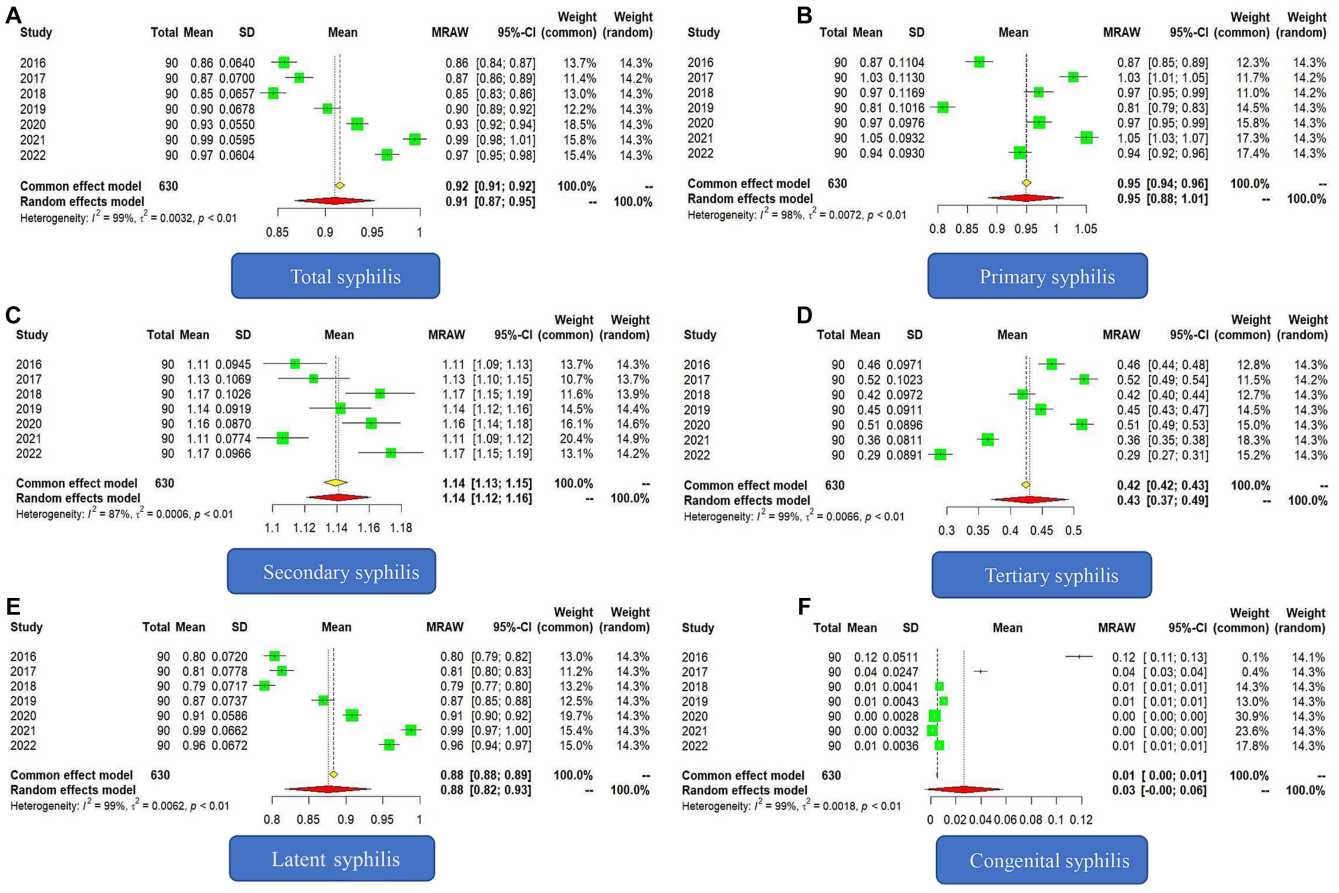
Forest plot of the scaling exponent of syphilis incidence from 2016 to 2022. **(A)** Forest plot of total syphilis. **(B)** Forest plot of primary syphilis. **(C)** Forest plot of secondary syphilis. **(D)** Forest plot of tertiary syphilis. **(E)** Forest plot of latent syphilis. **(F)** Forest plot of congenital syphilis.

From 2016 to 2022, the scaling exponent of the total syphilis incidence showed an upward trend but did not exceed 1. The scaling exponents for these 7 years were 0.86 (95% CI: 0.84 to 0.87), 0.87 (95% CI: 0.86 to 0.89), 0.85 (95% CI: 0.83 to 0.86), 0.90 (95% CI: 0.89 to 0.92), 0.93 (95% CI: 0.92 to 0.94), 0.99 (95% CI: 0.98 to 1.01) and 0.97 (95% CI: 0.95 to 0.98), respectively. The Higgins I2 value of 99% and the *Q* statistic value of 444.91(*p* < 0.001) indicated that there was heterogeneity in the scaling exponents of each year. Therefore, the results of the random effects model were used; that is, the combined scaling exponent was 0.91 (95% CI: 0.87 to 0.95) (Figure 1A).

The scaling exponent of the incidence of primary syphilis fluctuated from 2016 to 2022, with the scaling exponent of all other years approaching 1, except for 2016 and 2019, for which the scaling exponents were significantly lower than 1. The Higgins *I^2^* value was 98%, and the *Q* statistic value was 381.23 (*p* < 0.001). The combined scaling exponent of the random effects model was 0.95 (95% CI: 0.88 to 1.01) (Figure 1B).

Starting in 2016, the scaling exponent of secondary syphilis in each year was greater than 1, showing a superlinear relationship with population growth. The Higgins *I^2^* value was 87%, and *Q* statistic value was 47.88(*p* < 0.001). The combined scaling exponent of the random effects model was 1.14 (95% CI: 1.12 to 1.16) (Figure 1C).

From 2016 to 2022, the scaling exponents of tertiary syphilis were all less than 1, and there was a downward trend in the past 2 years. Unlike secondary syphilis, the relationship between the incidence of tertiary syphilis and population size exhibited a sublinear relationship. The Higgins *I^2^* value was 99%, the *Q* statistic value was 435.87(*p* < 0.001). The combined scaling exponent of the random effects model was 0.43 (95% CI: 0.37 to 0.49) (Figure 1D).

In terms of latent syphilis, the Higgins *I^2^* value was 99%, the *Q* statistic value was 696.93(*p* < 0.001), and the combined scaling exponent of the random effects model was 0.88 (95% CI: 0.82 to 0.93). In general, there was a sublinear relationship between the incidence of latent syphilis and population size, but starting in 2016, the scaling exponent showed an upward trend and was close to 1 (Figure 1E).

The Higgins *I^2^* value of congenital syphilis was 99%, and the *Q* statistic value was 963.32 (*p* < 0.001). The combined scaling exponent of the random effects model was 0.0264 (95% CI: −0.0047 to 0.0575). Because the confidence interval contained 0, the scaling exponent was not statistically significant (Figure 1F).

### Multivariate time series analysis

All 90 counties in Zhejiang Province have been affected by the syphilis epidemic. The multivariate time series model was constructed based on monthly data from 2016 to 2022 to identify the heterogeneity of spatiotemporal transmission. In the first step, we built three models to ascertain the spatial contiguity weights matrix based on the assumption of a negative binomial distribution of the incidence, which included the first-order neighbourhood model, the second-order neighbourhood model and the power law model. The AICs of the three models were 50,159.39, 50,150.15 and 50,119.00, respectively. The power law model was better than the other two models. In the second step, we built two power law models to select the distribution of the incidence. Models with a negative binomial distribution and a Poisson distribution were compared. The AICs of these two models were 50,119.00 and 54,795.11, which suggested that the model with the assumption of a negative binomial distribution was better.

The overall average effect values of the endemic component, spatiotemporal component and autoregressive component for all counties were 0.24, 0.035, and 0.72, respectively. The autoregressive component had the most significant impact on syphilis, followed by the endemic component, and the spatiotemporal component had the least impact.

**Figure 2:**
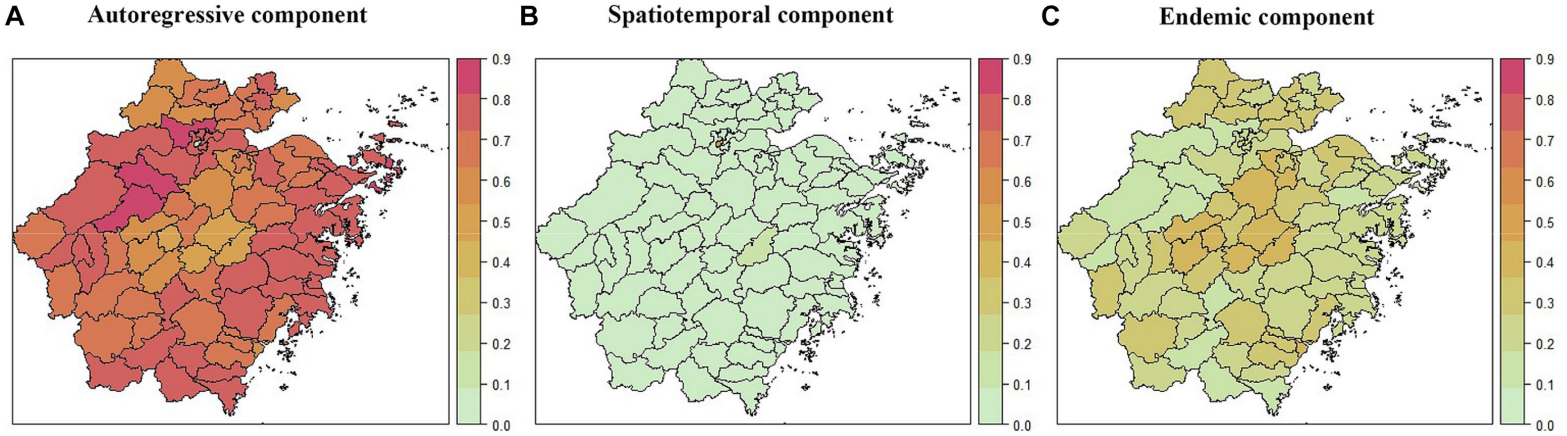
The district-specific fitted component of syphilis in Zhejiang Province, China, 2016–2022. **(A)** The autoregressive component at the county level. **(B)** The spatiotemporal component at the county level. **(C)** The endemic component at the county level.

There was obvious heterogeneity across counties in the average values of the endemic component and the autoregressive component. However, the effects of the spatiotemporal component did not differ significantly among counties. For the autoregressive component, the maximum value was 0.86 in Tonglu County, and the minimum value was 0.28 in Xihu Landscape County (Supplementary Box S3). The values of the autoregressive component for most counties were greater than 0.7, and the 10 counties with the lowest values were Longwan County (0.6101), Deqing County (0.6089), Keqiao County (0.6077), Lanxi County (0.6075), Zhuji County (0.6040), Pan’an County (0.5512), Jindong County (0.5508), Dongyang County (0.5465), Yongkang County (0.5417) and Xihu Landscape County. As shown in the map, most of the counties with low values of the autoregressive component are located in the central area of Zhejiang Province, where Shaoxing city and Jinhua city are located (Figure 2A). For the endemic component, the top 10 counties with the highest values were Dongyang County (0.4234), Jindong County (0.4208), Yongkang County (0.4171), Zhuji County (0.3741), Keqiao County (0.3738), Lanxi County (0.3692), Wucheng County (0.3544), Pan’an County (0.3514), Longwan County (0.3514) and Pinghu County (0.3447). Among the top 10 counties mentioned above, eight are located in the central region of Zhejiang Province, and among them, Keqiao County, Lanxi County, Zhuji County, Pan’an County, Jindong County, Dongyang County, and Yongkang County were coincidentally the counties with the lowest values of the autoregressive component (Figure 2B). For most counties, the values of the spatiotemporal component were less than 0.1, which means that the interpretable percentage of incidence of syphilis driven by spatiotemporal transmission in these areas was less than 10%. Only two counties had spatiotemporal component values greater than 0.1: Xihu Landscape County (0.5460) and Shengsi County (0.1311) (Figure 2C).

**Figure 3:**
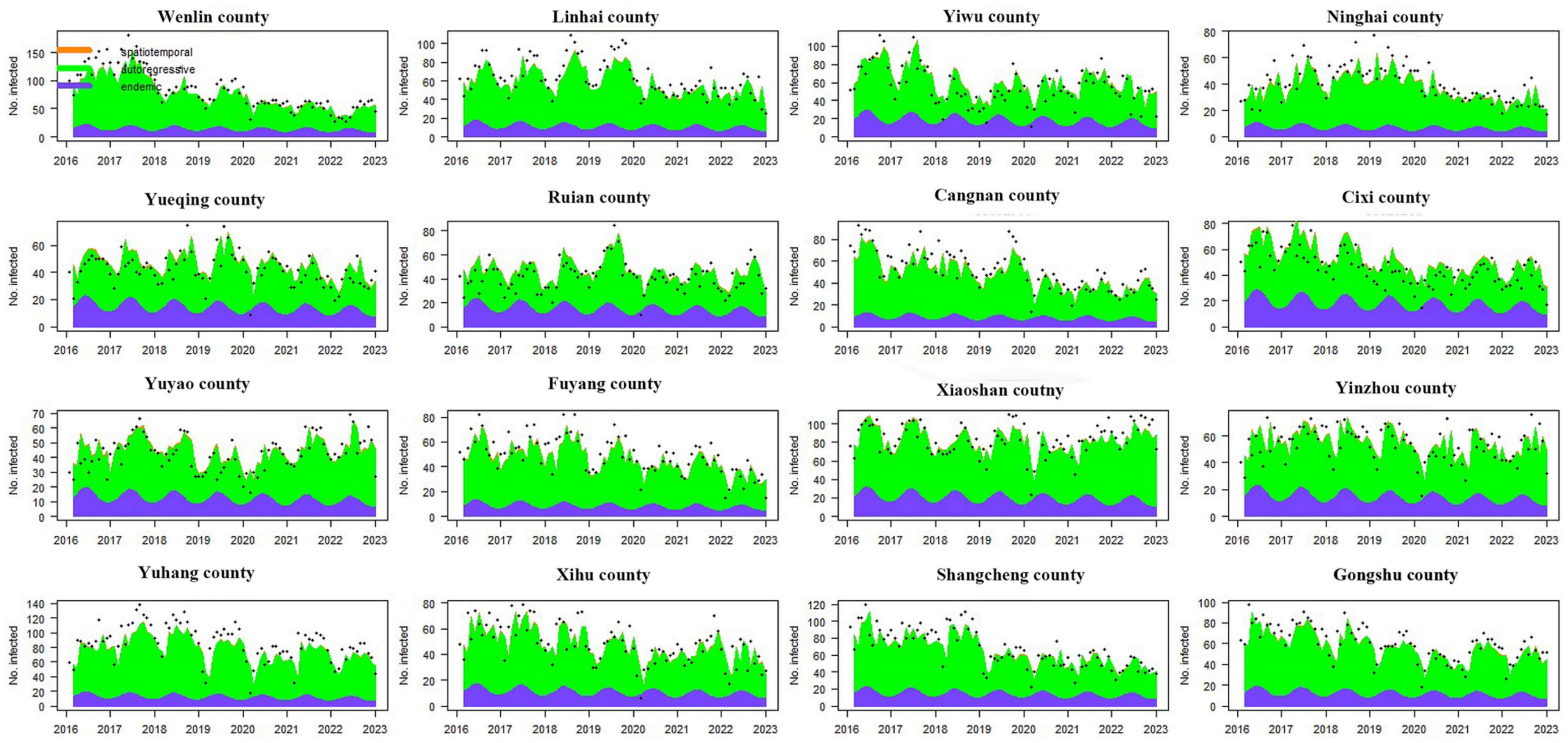
The time series plot of fitted components for the top 16 counties with the highest incidence. The black dots represent the monthly incidence, the blue area shows the endemic component, the green area shows the autoregressive component, and the orange area corresponds to the spatiotemporal component. The region codes represent Wenlin County, Linhai County, Yiwu County, Ninghai County, Yueqing County, Ruian County, Cangnan County, Cixi County, Yuyao County, Fuyang County, Xiaoshan County, Yinzhou County, Yuhang County, Xihu County, Shangcheng County, and Gongshu County from left to right and from top to bottom.

The time-varying importance of the three components in the high-incidence areas (the top 16 counties with the highest incidence) was plotted along with the observed counts. Figure 3 shows that the incidence of syphilis in these regions was mainly driven by the endemic component and the autoregressive component, where the characteristics of the endemic component were homogeneous, while the autoregressive component showed significant heterogeneity. From 2016 to 2022, the endemic component of each county showed obvious seasonal changes, with peaks from May to September and valleys from November to February of the next year. At the same time, the contribution of the endemic component in each year was similar. The autoregressive component of each county also exhibited similar seasonal characteristics, but the trends in each county varied greatly. In Wenling County, Linhai County, Ninghai County, Cangnan County, Fuyang County, Xihu County, Shangcheng County and Gongshu County, the effect of the autoregressive component showed an annual downward trend, while this component in other counties had no such characteristics in the past 7 years. For the above 16 counties, the impact of the spatiotemporal component was almost negligible (Figure 3).

**Figure 4:**
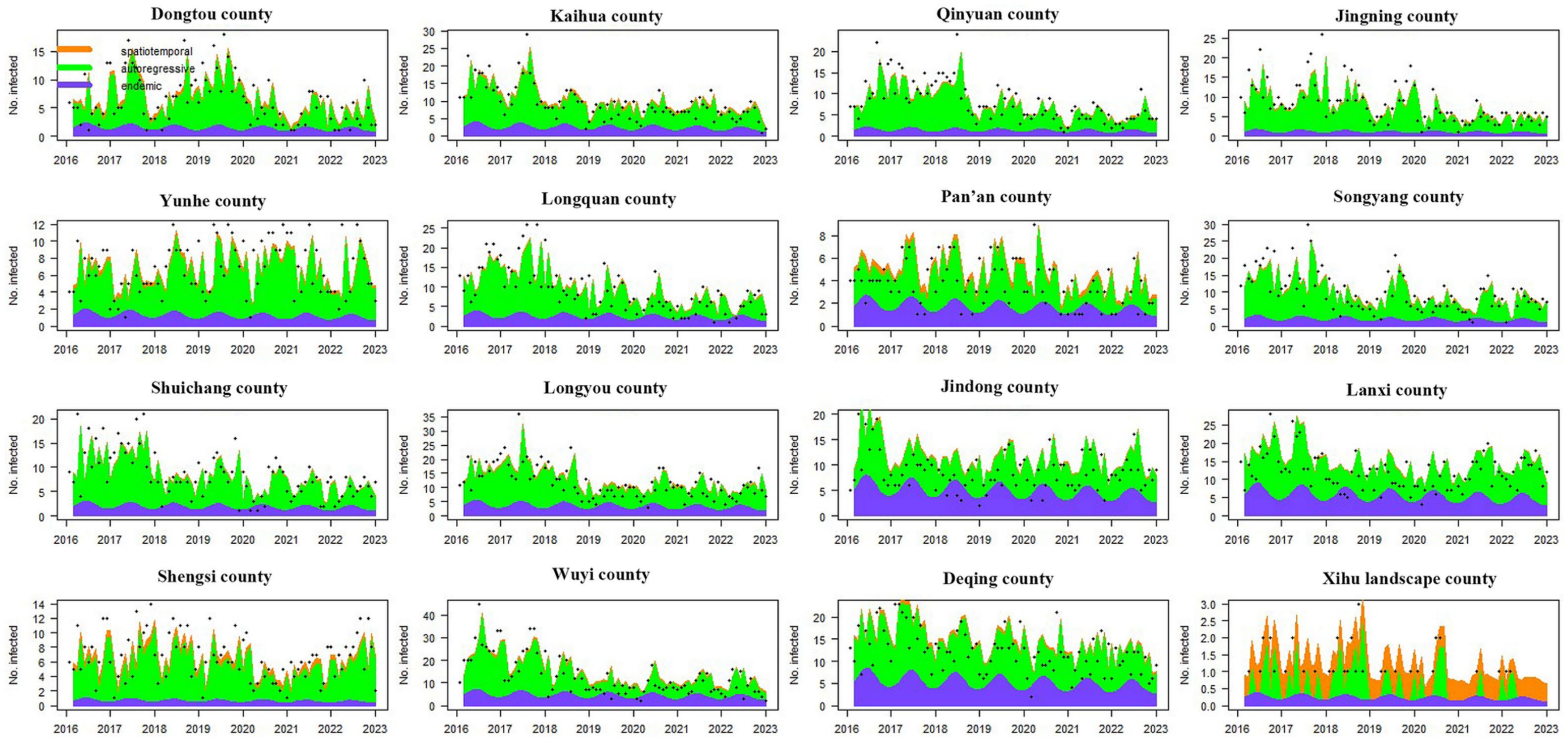
The time series plot of fitted components for the top 16 counties with the lowest incidence. The black dots represent the monthly incidence, the blue area shows the endemic component, the green area shows the autoregressive component, and the orange area corresponds to the spatiotemporal component. The region codes represent Dongtou County, Kaihua County, Qinyuan County, Jingning County, Yunhe County, Longquan County, Pan’an County, Songyang County, Shuichang County, Longyou County, Jindong County, Lanxi County, Shengsi County, Wuyi County, Deqing County, and Xihu Landscape County from left to right and from top to bottom.

For the 16 counties with the lowest incidence, there were considerable differences in the time-varying effects of the three components. Similar to the high-incidence counties, the endemic component of low-incidence counties also showed a stable seasonal cycle. In addition to Xihu Landscape County, the epidemic situation in other counties was mainly driven by the autoregressive component. Furthermore, the autoregressive component in Kaihua County, Qinyuan County, Jingning County, Longquan County, Songyang County, Suichang County, Longyou County, and Wuyi County also exhibited a downward trend. Unlike in other counties, the syphilis epidemic in Xihu Landscape County was mainly influenced by the spatiotemporal component, which means that the epidemic in its surrounding areas made a major contribution to the development of the epidemic in Xihu Landscape County. In addition, we found that Dongtou County, Kaihua County, Yunhe County, Pan’an County and Shengsi County were affected by the spatiotemporal component to a certain extent (see Figure 4).

## Discussion

The downward trend of the syphilis epidemic is basically consistent with previous research results in Zhejiang Province (11). Our study showed that from 2016 to 2022, the reported incidence rate of all types of syphilis in Zhejiang Province declined, which was closely related to a variety of factors. Among all possible relevant factors, the most important was related to the strict implementation of policies such as the Zhejiang Provincial Plan for the Prevention and Control of Syphilis (2011–2020) and the Implementation Rules of the Zhejiang Provincial Measures for the Prevention and Control of Sexually Transmitted Diseases. In the past 10 years, Zhejiang Province has continued to implement standardized diagnosis and treatment services for sexually transmitted diseases (STDs) and strengthened active syphilis screening, high-risk behaviour intervention and other comprehensive prevention and control measures for STD patients, AIDS counselling and testing and drug maintenance treatment for outpatients and those at risk of AIDS/STDs so that syphilis patients can receive timely and standardized treatment and the spread of syphilis can be effectively controlled (36, 37). According to the values of DISO, the joinpoint regression model of primary syphilis, secondary syphilis, tertiary syphilis and congenital syphilis have good fitting performance in simulating the annual incidence rate (33). However, the DISO of total syphilis and latent syphilis indicated that the performance of the joinpoint regression model for these two types of syphilis were relatively poor. The main reason is that the incidence rate of these two types of syphilis shown a fluctuating downward trend rather than a continuous downward trend, so the fitting error was obvious in some years.

The World Health Organization launched a plan to eliminate mother-to-child transmission of syphilis in 2007 and reported in 2017 that 10 countries, including Thailand and Cuba, successfully eliminated congenital syphilis (38). In the Syphilis Prevention and Control Plan issued in 2010, China proposed that by 2020, the reported incidence rate of congenital syphilis should be below 15/100,000 live births. The average reported incidence rate of congenital syphilis in Zhejiang Province in the past 5 years was 1.3/100,000, which is better than the target required by policies, mainly due to integrated prevention and control strategies such as increased prenatal screening coverage, increased treatment completion, and earlier prenatal screening (6, 39). Nevertheless, there are still reports of congenital syphilis in Zhejiang Province every year, and the prevention of mother-to-child syphilis is particularly problematic for mobile populations, people in remote areas, and vulnerable groups in terms of accessibility and timeliness. Furthermore, the fear of stigma prevents pregnant women from seeking testing and treatment, which aggravates the “stealthy” spread of the disease in the population (40). Therefore, it is necessary to improve premarital and prenatal syphilis screening, including the use of self-testing reagents, to strengthen syphilis monitoring for women from migrant populations, achieve early detection and treatment, effectively control the mother-to-child transmission of syphilis, and reduce the occurrence of congenital syphilis and adverse pregnancy outcomes related to syphilis.

Another important reason for the decline in the reported incidence rate of syphilis in Zhejiang Province was that the quality verification of STD reports was strengthened, which has improved the accuracy of syphilis diagnosis and reporting while reducing the frequency of repeated reporting. According to previous studies in Zhejiang Province, there are serious duplicate reports of syphilis cases, which misrepresents the true number of syphilis cases. The duplicate report rate from 2016 to 2018 was between 16.63% and 19.93%, and the duplicate report rate from 2019 to 2020 decreased but still reached as high as 9.37% (41). This suggests that it is necessary to further check for duplicates at medical institutions.

Overall, the incidence level of syphilis was highly correlated with population size, which is similar to previous research results (12). Previous studies have shown that the scaling relationship between population size and sexually transmitted diseases such as HIV often exhibits a superlinear correlation (19, 21). However, unlike previous studies, our study found that the scaling exponent had both temporal trend characteristics and significant heterogeneity in the association between each type of syphilis and population size. From the perspective of the merger effect of scaling law, the overall incidence of syphilis had a sublinear relationship with population size. Furthermore, the scaling law values from 2016 to 2022 showed a significant upward trend, with 2016 to 2020 showing a sublinear feature, while 2021 and 2022 showed a linear relationship. Due to the multiple stages of syphilis, the epidemiological connotations represented by each stage of syphilis also vary greatly. Usually, primary, secondary, and congenital syphilis can all be diagnosed as new cases, while tertiary syphilis with a course of more than 2 years often represents a previous infection. Cases of latent syphilis have no clinical symptoms or signs and are often detected through screening, making it difficult to determine whether it is a new infection. Therefore, analysis of the overall level of syphilis incidence alone cannot accurately express the impact of factors such as population size on the trend of syphilis incidence. To avoid bias, our study further explored the scaling relationship between syphilis in each stage and population size, which is also the largest difference from previous studies.

The scaling exponent of primary syphilis was close to 1, indicating a linear relationship between the incidence of primary syphilis and population size. This means that the growth rate of primary syphilis was almost consistent in regions with different population sizes. According to reports in the literature, there are considerable differences in the driving factors of syphilis incidence in different regions. In areas with smaller populations, the economy is often underdeveloped, leading to poorer public health facilities, lower levels of education, and poorer health awareness, thus facilitating the spread of syphilis (42). However, in large cities, rapid and major population movement, more complex social communication networks and a higher proportion of sexually active individuals, such as men who have sex with men (MSM), have led to a high incidence of sexually transmitted diseases, such as syphilis (11). The scaling exponent of secondary syphilis presented an interesting result, with a superlinear correlation with population size. Moreover, this superlinear relationship was very stable across all years. This situation means that in large cities with larger populations, the growth rate of secondary syphilis with population changes was faster, while in small cities or rural areas with smaller populations, the growth rate of secondary syphilis was slower. We consider that the main reason for this phenomenon is the difference in diagnostic accuracy between primary and secondary syphilis in different regions. In large cities, the diagnostic accuracy of syphilis is higher, and there are fewer misclassifications of syphilis in each stage. At the same time, large cities may also have wider monitoring coverage (43). In small cities, there may be a relatively high incidence of misdiagnosis of secondary syphilis as primary syphilis. Furthermore, this misdiagnosis can also affect the occurrence of tertiary syphilis. This was confirmed by the scaling exponent of tertiary syphilis. In the 7 years studied, the scale relationship between tertiary syphilis and population size showed a sublinear relationship, which indicated that the growth rate of tertiary syphilis in small cities or rural areas was faster. The most likely reason for this situation is that the missed diagnosis or misdiagnosis of secondary syphilis has to some extent delayed the accurate treatment of secondary syphilis, leading to more cases progressing to tertiary syphilis. This also suggests that it is necessary to improve the accuracy of syphilis diagnosis and the standardization of treatment in small cities and rural areas. As is well known, the reporting of latent syphilis is closely related to the level of antibody screening, which cannot be used to reflect the new incidence trend of syphilis but can only represent the overall syphilis situation, including history and current status. There are two characteristics of the scaling exponent of latent syphilis: first, the overall scale relationship exhibited a sublinear feature, and second, as the years passed, the scale relationship changed from a sublinear to a linear relationship. A study showed that 78.83% of latent syphilis patients were detected in the departments of surgery and gynaecology (44). With the implementation of the Syphilis Prevention and Control Plan, medical institutions at all levels have continuously strengthened syphilis screening for hospitalized patients, surgical patients, and pregnant women, resulting in an increasing number of reported cases of latent syphilis. Compared to large cities, the level of diagnosis and treatment in small cities and rural areas is lower, with more nonstandard diagnosis and treatment (such as small clinic visits). Some syphilis cases have not been accurately diagnosed or reported in the past. Therefore, in the early stages of prevention and control planning implementation, the growth rate of latent syphilis discovered through screening was higher in small cities and rural areas. Subsequently, with the improvement of diagnostic and treatment standards in small cities and rural areas, as well as the reduction of historical case detection, the growth rate of latent syphilis in regions with different population sizes gradually converged. Due to the small number of congenital syphilis cases, there was no scaling relationship between population size. This also suggests that the incidence of congenital syphilis in Zhejiang Province may be sporadic.

From the perspective of the spatiotemporal driving factors that affected the onset of syphilis, the most important driving factors were the autoregressive component, which means that the early syphilis epidemic played a major role in the impact of subsequent cases. Overall, it can explain 72% of the epidemic in the province. One possible reason for this situation is that some medical institutions do not yet have the two types of serological tests for syphilis diagnosis (syphilis spirochete serological tests and non-syphilis spirochete serological tests). The syphilis screening conducted for high-risk groups still faces issues such as insufficient capacity at prevention and control institutions, insufficient technical skills of medical personnel, and difficulty in identifying target populations, which have affected the coverage rate of screening, leading to missed diagnoses of some cases. Another reason may be the lack of benzylpenicillin in some regions or medical institutions or the insufficient understanding of recommended protocols in the guidelines by medical staff, which has resulted in some syphilis-infected individuals not receiving standardized treatment. The above two factors suggest that timely detection of cases and correct treatment after detection play important roles in controlling the development of the current syphilis epidemic.

In addition to autoregressive components, we found that the epidemic in some regions was also affected by endemic components. This suggests that these regions may not pay enough attention or invest sufficiently in the prevention and control of sexually transmitted diseases such as syphilis, the participation of relevant departments is not sufficient, and the cooperation between departments is poor, which affects the effective implementation of syphilis prevention and control measures.

Our results were similar to a previous study in Japan in which endemic components including use of mobile dating software and number of sex trade shops per prefectural population were positively correlated with incidence rate (45). On the other hand, it may also suggest that in these areas with high endemic component values, the health literacy of the population in terms of self-prevention is relatively low. These populations may engage in more commercial and high-risk sexual activities, and there are relatively more cases of delayed treatment and nonstandard treatment. We found similar factors in a study in Brazil which mentioned that the incidence of syphilis was associated with municipalities with poor sanitary conditions, lower proportion of pregnant teenagers, and under 8 years of schooling (46). Furthermore, it should be noted that in recent years, many studies have found that older adult men who are physically healthy, have a low level of education, are economically stable, and are widowed or divorced are at high risk of engaging in illegal prostitution. It is recommended to strengthen the promotion of sexually transmitted disease prevention and control in the older adult population and actively guide them to participate in healthy entertainment habits (32). Although the epidemic situation in most areas was mainly affected by the autoregressive component and endemic component, there were still a few areas with obvious spatiotemporal dissemination characteristics, which means that the epidemic in these regions was greatly affected by imported cases from surrounding regions. This suggests that for the prevention and control of syphilis in Xihu Landscape County and Shengsi County, it is necessary to work together with surrounding areas to improve control efficiency.

In terms of spatiotemporal driving factors, we found that both the autoregressive component and endemic component showed obvious seasonal and cyclical characteristics. Almost all counties showed a summer peak and winter trough, and these seasonal characteristics continued every year. The seasonality of this driving factor determined the seasonal high incidence of syphilis. There are two possible reasons for the seasonality of the syphilis epidemic. First, a warm climate may affect hormone levels, thereby promoting an increase in sexual activity. Second, during the Spring Festival in winter, people may have reduced willingness to seek medical treatment to celebrate festivals and gatherings (11, 44). The development trend of autoregressive factors in counties showed significant heterogeneity. This suggests that counties that showed a downward trend may gradually control the epidemic through the diagnosis, treatment, and management of early cases. In areas where the autoregressive factor was consistently high or even rising, further prevention of infection and transmission among high-risk populations is needed; at the same time, it is necessary to strengthen efforts to block transmission from high-risk populations to the general population and prevent mother-to-child transmission.

Several limitations should be noted in our study. First, due to the current reporting system consisting of passive monitoring, some undetected cases could not be included in the analysis. At the same time, there were also cases of duplicate reporting and misclassification in the reporting system, which to some extent may have led to data bias. Second, although scaling law is generally applicable to every urban system and is independent of factors such as urban geographical location, culture, and economic development, there is still some controversy over the universality of scaling law. One is the instability of scaling laws within different urban ranges, and another is the applicability of linear regression models under dual logarithmic conditions. These factors may have affected the accuracy of the scaling exponent. Third, due to insufficient data, the influence of covariates on the scaling law has not been corrected, which needs to be further improved and verified in future research. Fourth, we were unable to collect information on covariates other than population in each county, resulting in a lack of precise analysis of the specific factors affecting the endemic components. Despite the shortcomings mentioned above, the research results still provide useful references for future syphilis epidemic prevention and control.

## Conclusion

The scaling exponent had both a temporal trend and significant heterogeneity in the association between each type of syphilis and population size. Primary syphilis and latent syphilis exhibited a linear pattern, secondary syphilis presented a superlinear pattern, and tertiary syphilis exhibited a sublinear pattern. This suggested that further prevention of infection and transmission among high-risk populations is necessary. Improvement of diagnostic accuracy and the standardization of treatment in small cities and rural area is also needed. In the analysis of the multivariate time series model, the autoregressive components and the endemic components were the main driving factors. Targeted prevention and control strategies must be developed based on the main driving factors of the epidemic in each county.

## Data availability statement

The original contributions presented in the study are included in the article/Supplementary material, further inquiries can be directed to the corresponding authors.

## Ethics statement

The studies involving humans were approved by Ethics Committee of the Zhejiang Provincial Center for Disease Control and Prevention. The studies were conducted in accordance with the local legislation and institutional requirements. Written informed consent for participation in this study was provided by the participants’ legal guardians/next of kin.

## Author contributions

HW: Formal analysis, Writing – original draft. MX: Writing – review & editing. CW: Investigation, Writing – review & editing. QL: Investigation, Writing – review & editing. ZD: Data curation, Writing – review & editing. XW: Validation, Writing – review & editing. TF: Investigation, Writing – review & editing. KY: Validation, Writing – review & editing. JL: Funding acquisition, Writing – review & editing.
