# Supplementary material for: Scaling law characteristics and spatiotemporal multicomponent analysis of syphilis from 2016 to 2022 in Zhejiang Province, China
Source: Front Public Health. 2023 Oct 25;11:1275551. doi: 10.3389/fpubh.2023.1275551 (PMC10642232; doi:10.3389/fpubh.2023.1275551)
Supplement: Supplementary file 2 [file Data_Sheet_1.docx]

**Supporting information**

File S1. Database of the monthly distribution of syphilis cases in Zhejiang Province from 2016 to 2022. (EXCEL)

Box 1. The main result of joinpoint regression

| Types of syphilis | *p* value of null hypothesis for 0 joinpoints | Joinpoints | Segment | APC(%) | Confidence interval of APC(%) | *p* value of APC(%) |
| --- | --- | --- | --- | --- | --- | --- |
| Total syphilis | 0.33 | 0 | 1 | -9.60 | (-12.90,-6.10) | <0.001 |
| Primary syphilis | 0.60 | 0 | 1 | -21.70 | (-26.70,-16.30) | <0.001 |
| Secondry syphilis | 0.49 | 0 | 1 | -16.80 | (-20.30,-13.30) | <0.001 |
| Tertiary syphilis | 0.64 | 0 | 1 | -8.70 | (-11.30,-6.00) | <0.001 |
| Congenital syphilis | 0.09 | 0 | 1 | -39.00 | (-49.30,-26.60) | 0.001 |
| Latent syphilis | 0.14 | 0 | 1 | -7.10 | (-11.20,-2.80) | 0.008 |

Box 2. Evaluation of simulation of joinpoint regression for all types of syphilis

| Index | Total syphilis | Primary syphilis | Secondry syphilis | Tertiary syphilis | Congenital syphilis | Latent syphilis |
| --- | --- | --- | --- | --- | --- | --- |
| CC | 0.96 | 0.98 | 0.98 | 0.97 | 0.97 | 0.90 |
| NAE | 0.99 | 0.07 | 0.04 | 0.002 | 0.001 | 1.00 |
| NRMSE | 1.00 | 0.11 | 0.09 | 0.005 | 0.002 | 0.98 |
| DISO | 1.41 | 0.13 | 0.10 | 0.03 | 0.03 | 1.40 |

Box 3. The top ten counties with the highest component of syphilis

| county | autoregressive component | county | spatio-temporal component | county | endemic component |
| --- | --- | --- | --- | --- | --- |
| Tonglu | 0.8613 | Xihu landscap | 0.5460 | Dongyang | 0.4234 |
| Putuo | 0.8601 | Shengsi | 0.1311 | Jindong | 0.4208 |
| Yuhang | 0.8369 | Pan’an | 0.0974 | Yongkang | 0.4171 |
| Jiande | 0.8363 | Dongtou | 0.0925 | Zhuji | 0.3741 |
| Changshan | 0.8201 | Yunhe | 0.0746 | Keqiao | 0.3738 |
| Wenlin | 0.8171 | Kaihua | 0.0719 | Lanxi | 0.3692 |
| Cangnan | 0.8158 | Wuyi | 0.0598 | Wucheng | 0.3544 |
| Linan | 0.8156 | Jinning | 0.0552 | Pan’an | 0.3514 |
| Taishun | 0.8151 | Daishan | 0.0533 | Longwan | 0.3514 |
| Linhai | 0.8118 | Songyang | 0.0515 | Pinghu | 0.3447 |
